# Oral and Non-Oral Cholesterol-Lowering Drugs with PCSK9 and Other Biomolecules as Targets: Present Status and Future Prospects

**DOI:** 10.3390/biom15040468

**Published:** 2025-03-22

**Authors:** Ajoy Basak

**Affiliations:** Department of Pathology and Laboratory Medicine, Faculty of Medicine, University of Ottawa, 451 Smyth Road, Ottawa, ON K1H 8M5, Canada; abasa2@uottawa.ca or Ajoybasak1@gmail.com; Tel.: +1-613-878-7043

**Keywords:** Proprotein Convertase Subtilisin Kexin9, LDL-receptor, LDL-cholesterol, EGF-A domain, PCSK9 inhibitors, monoclonal antibody, cholesterol-lowering drugs, bile acid sequestrants, statins, MK0616, AZD0780

## Abstract

The accumulation of high levels of cholesterol associated with low-density lipoprotein (LDL) in the bloodstream is the key risk factor for stroke and cardiovascular diseases. Therefore, reducing the concentration of LDL-cholesterol in the blood and maintaining it at an optimum level are vital especially for hypercholesterolemic individuals and cardiovascular patients. Thus, the study of cholesterol management and regulation in the physiological system has drawn significant attention from researchers across the globe. This led to the discovery of several cholesterol-lowering drugs which have been approved for administration either via oral or non-oral routes. Owing to the high comfort level, reduced or no pain, and fewer side effects with oral administration, more focus has been directed towards the development of oral-based cholesterol-lowering drugs. The other modes of administration such as intravenous or intramuscular injections are complex and sometimes painful and less tolerable. Therefore, there was a significant interest to develop oral drugs targeting PCSK9. In fact, some progress has been accomplished in recent years. This review provides an overview of the existing cholesterol-lowering drugs, and the progress made so far with oral-based PCSK9 drugs for lowering LDL-cholesterol. The review is presented in several sections highlighting the molecular targets, the individual drugs, and the modes of administration, with a focus on the oral route.

## 1. Introduction

### 1.1. Cholesterol Background and Function

Cholesterol is a key steroid biomolecule that plays many essential functional roles in biology. The word was derived from chole (bile), stereos (solid), and -ol for the alcohol group that it contains. This lipophilic fatty material helps to build cell membranes, contributes to its structural make up and integrity, and controls their fluidity. It is also vital for the production of steroid hormones such as cortisol, aldosterone, and adrenal androgens, as well as sex hormones like androgens (e.g., testosterone), estrogens (e.g., estradiol), and progestogens (e.g., progesterone) [1]. Cholesterol is also required for the synthesis of vitamin D in the body. Additionally, it helps in the digestion process. In fact, it is a constituent of bile acids which are responsible for the absorption of fat-soluble vitamins such as vitamins A, D, E, and K. Moreover, cholesterol is required in maintaining cellular homeostasis [2,3]. It is synthesized by the liver in the body, although it can also be supplied externally via the consumption of foods like dairy products and meats. However, cholesterol must be maintained at an optimal level for the healthy function of the body. Its imbalance (either excess or deficiency) can cause serious disorders or diseases in the body [4,5,6].

### 1.2. Cholesterol Level and Carrier Lipoproteins

Due to its fatty nature, cholesterol is insoluble in aqueous media. Once synthesized in the liver, cholesterol, along with other more hydrophobic lipids such as triglycerides (TGs) and cholesterol esters (CEs), is carried and transported through the bloodstream in the form of micelles. These spherical micelles are composed of TGs and CEs making the central core which is surrounded by a monolayer of phospholipids integrated with exchangeable proteins called apolipoproteins and free cholesterol. This whole complex package is defined as a lipoprotein particle which can be of several types. These include LDL (low-density lipoprotein), VLDL (very low-density lipoprotein), IDL (intermediate-density lipoprotein), HDL (high-density lipoprotein), and chylomicron (the least dense particle). As a complex with the LDL particle, cholesterol is transferred to the interior of the cells via a specific receptor present on the cell surface. This LDL-associated cholesterol, known as “bad cholesterol,” in lay terms, can accumulate as a plaque inside the arteries of blood vessels, thereby making them too narrow for blood to flow freely. This condition is known as “atherosclerosis”. Like the LDL, the VLDL particle also carries cholesterol from the liver to the tissues but to a much lesser extent. However, it primarily carries TGs. Finally, the IDL particle, which is generated from VLDL following the loss of triglyceride chains, is a transitional or intermediate lipoprotein between VLDL and LDL [7,8]. It is either removed by the liver or converted to LDL. The HDL-cholesterol is commonly known in lay terms as “good cholesterol” since here excess cholesterol is transported back to the liver for degradation and ultimate elimination from the body. This leads to the downregulation of cholesterol in the bloodstream [9,10].

The lipoproteins described above are classified according to their size or density and are designated accordingly. They regulate cholesterol levels in the bloodstream. It is fundamental that cholesterol homeostasis is properly maintained in the body for normal cellular and systemic activities. Any disturbance or alteration in cholesterol levels underlies not only cardiovascular diseases but also neurodegenerative diseases and cancers [11]. The cellular cholesterol level is highly dependent on the relative amounts of LDL-/VLDL- and HDL-cholesterols in the circulation system and their functional activities. Any deviations from the optimum levels of these two forms of cholesterol will lead to hyper- (excess cholesterol) or hypo- (deficient in cholesterol) cholesterolemic conditions, leading to pathologic outcomes. Familial hypercholesterolemia, which leads to the accumulation of excess LDL-C in the bloodstream, represents the highest risk factor for congenital heart disease (CHD) and stroke [12]. It is caused not only by high LDL-cholesterol and low HDL-cholesterol conditions but also by ApoE mutations and the accumulation of chylomicron remnants in the blood [13].

### 1.3. Cholesterol Biosynthesis and Dietary Sources

**Figure 1:**
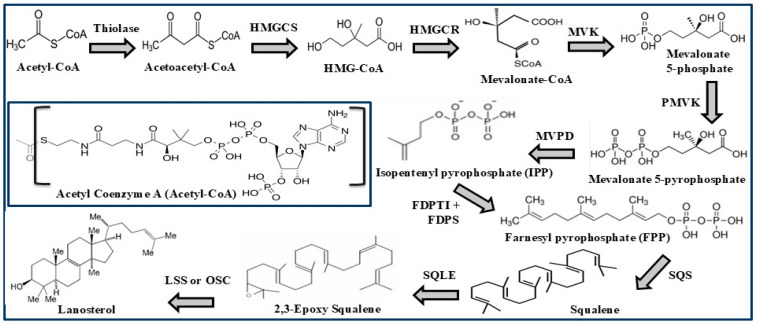
Biosynthesis of cholesterol in the liver showing the initial steps leading to the intermediate Lanosterol. Lanosterol is then converted to cholesterol via Kandutsch–Russell or Bloch parallel pathways as shown in Figure 2. HMG=3-Hydroxy 3-Methyl Glutaryl; HMGCS=HMG-CoA synthase; HMGCR=HMG-CoA reductase; MVK=Mevalonate kinase; PMVK=Phospho mevalonate kinase; MVPD=Mevalonate 5-pyrophosphate decarboxylase; FDPTI=Farnesyl diphosphate isomerase; FDPS=Farnesyl diphosphate synthase; SQS=Squalene synthase; SQLE=Squalene epoxidase; LSS= Lanosterol synthase; OSC=Oxido squalene cyclase.

**Figure 2:**
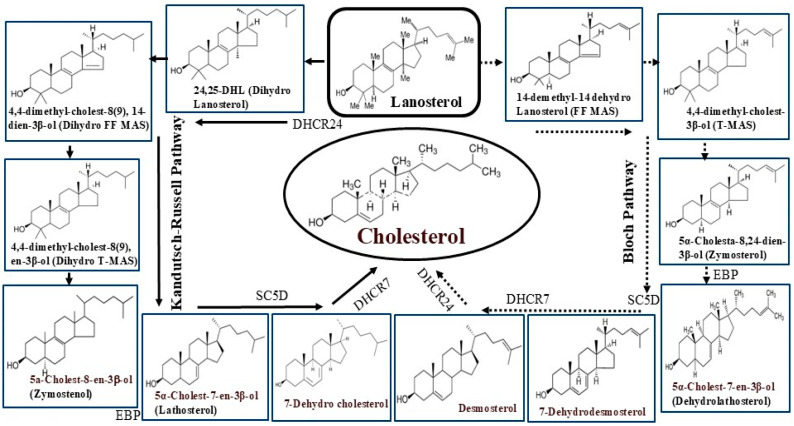
Post-lanosterol biosynthesis of cholesterol via Kandutsch–Russell (Left) and Bloch (Right) pathways which work in parallel. Selected enzymes implicated including the critical DHCR24 (Dehydrocholesterol reductase24) in the initial (K-R pathway) and final steps (Bloch pathway) are particularly significant. EBP: Emopamil binding protein (also called Hydroxy steroid isomerase); SC5D = Sterol C5 desaturase.

Most of the cholesterol requirement of the body is synthesized by the cells throughout the body but the primary site of cholesterol production is the liver where nearly 80% of the body’s total cholesterol needs are made. Following production, most cholesterol is packaged as a complex with lipoproteins which allow it to be transported via the bloodstream to various cells. The biosynthesis of cholesterol is complex in nature and is a multistep process. It starts with Acetyl-Coenzyme-A (Ac-CoA), which condenses with another molecule of Acetyl-CoA to produce Aceto-acetyl CoA. This then combines with a third molecule of Ac-CoA to generate 3-Hydroxy-3-methylglutaryl coenzyme A (HMG-CoA). HMG-CoA is then converted to mevalonate, a key molecule by the action of HMG-CoA reductase enzyme [13]. Mevalonate is then converted to cholesterol via squalene, lanosterol, and many other intermediate products. The entire route consists of two distinct, but interchangeable parallel pathways called Kandutsch–Russell (K-R) and Bloch pathways (Figure 1 and Figure 2), which divert from one another after the synthesis of lanosterol [14,15,16]. Various enzymes have been implicated in these pathways. These include the most crucial DHCR24 (Dehydrocholesterol reductase24) which plays a role in the initial step of the K-R pathway as well as in the final step of the Bloch pathway. It may also be mentioned that all the individual intermediate products of the Bloch pathway can be converted to the corresponding product in the K-R pathway via the action of DHCR24. In principle, cholesterol biosynthesis can be regulated by targeting any of the individual steps linked to either pathway. In fact, the step involving the conversion of HMG-CoA to mevalonate via the catalytic action of HMG-CoA reductase enzyme has been successfully exploited to prevent cholesterol synthesis.

Besides the biosynthesis of cholesterol within the body, one can also acquire cholesterol from external sources such as food. Dairy and meat products, as well as eggs and poultry, are the richest sources of cholesterol. Thus, dietary cholesterol intake can also be a contributary factor for cholesterol build-up in the body [17]. In fact, for many decades, it was well recognized that dietary cholesterol can cause the enhancement of blood cholesterol level. However, recent evidence and research suggest that many individuals can adapt to a high intake of cholesterol-rich food, yet it only has a marginal effect on their blood cholesterol levels. In general, hypercholesterolemia—a condition of high cholesterol—is primarily caused by the increased production of LDL-cholesterol or its reduced clearance but not by the increased intake of dietary cholesterol [18,19].

### 1.4. Cholesterol Pathology and Management

Extensive research studies have revealed that a high level of LDL-cholesterol in the bloodstream is an increased risk factor for developing cardiovascular diseases, such as heart attack and stroke. This is caused by the formation of atherosclerotic plaque within the arteries, leading to the obstruction of blood flow. In fact, it has been linked to coronary artery disease, aortic aneurysms, stroke, peripheral artery disease (PAD) (a common condition where narrowed arteries reduce blood flow), and others [20,21]. For this reason, the regular monitoring of LDL-cholesterol levels in the blood is recommended for individuals over 20 years of age even with no other risk factors.

It may be noted that both hyper- (excess) and hypo- (deficient) cholesterolemic conditions can lead to various pathologic disorders, with the former being more harmful to health. Hypocholesterolemia on the other hand, may cause anemia (low counts of red blood), liver and thyroid problems. It can also enhance hepatitis C infection [22]. A Japanese cohort study using ~12,300 adults within the age group of 40–69 years from 12 different locations of the country revealed that low cholesterol may be associated with mortality from stroke, heart disease, and cancer, but this finding is highly controversial [23]. However, several health risks of very low cholesterol levels in the blood have been documented and reviewed in [24]. Currently, there are more serious concerns with high rather than low levels of LDL-cholesterol in the blood. The data collected from World Health Organisation suggested that in 2008, the prevalence of high level of total cholesterol among the adults was nearly 39% (40% for females and 37% for males) [https://www.who.int/data/gho/indicator-metadata-registry/imr-details/3236; accessed on 11 March 2025]. This caused about 4.5% deaths from ischaemic heart disease/strokes and 2% of total DALYS (Disability-Adjusted Life Years) [25]. Thus, cholesterol control and management have become a crucial element in our fight against cardiovascular, stroke, and related diseases which are the leading cause of human mortality [26]. CVDs are responsible for the deaths of 20.5 million people worldwide, representing nearly one-third of all global deaths according to 2021 data [27]. As dietary cholesterol and lifestyle changes seem to have no or marginal effect on ultimate cholesterol build-up in the body, attention has shifted towards controlling cholesterol’s biosynthesis and it’s clearance from the body. This has resulted in the discovery of several drugs now commercially available for lowering LDL-cholesterol in the blood serum.

## 2. Biomolecules as Targets for Lowering Cholesterol

A few selected biomolecules including enzymes and proteins have been identified as potential targets for the development of cholesterol-lowering drugs. In addition, specific biological events associated with the gastrointestinal (GI) pathway have also been considered as potential targets for degrading or clearing cholesterol. These molecular targets, which led to the development of several cholesterol-lowering drugs, are discussed in the sections below.

### 2.1. HMG CoA Reductase (HMGCR)

**Figure 3:**
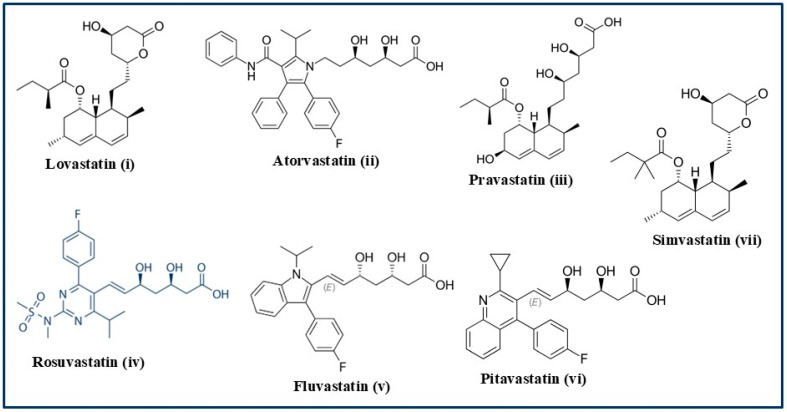
The chemical structures of seven known statin drugs approved for global use to lower LDL-cholesterol.

This key enzyme present in the liver has been implicated in the upstream event of the cholesterol biosynthesis pathway. It is, in fact, the rate-limiting step of the cholesterol biosynthesis pathway, which is why most inhibitors are developed to target this step. The effective inhibition of this enzyme leads to a massive compensatory upregulation of the expression of this enzyme in the Endoplasmic Reticulum (ER), which often leads to ER stress. Small-molecule promoters of HMGCR proteolytic degradation have been designed to address this [28]. HMGCR plays a crucial role in the conversion of HMG-CoA to mevalonate which is a precursor molecule of lanosterol. Lanosterol is the key intermediate for cholesterol synthesis via either the Kandutsch–Russell or the Bloch pathway, both leading to the production of cholesterol in the liver. Thus, blocking or inhibiting HMG-CoA reductase activity should be able to prevent the synthesis of cholesterol via either route [28]. Therefore, attention has been drawn to develop inhibitors against this enzyme. Extensive research worldwide has led to the discovery of the first HMG-CoA reductase inhibitor called Lovastatin, which was the first “statin” drug approved for lowering LDL-cholesterol and total cholesterol while promoting HDL-cholesterol [29]. The name “statin” comes from the Latin word “stare” meaning “remaining stand still”, as it blocks the rise in cholesterol levels. Statins are a group of cholesterol-reducing drugs that are classified as HMG-CoA reductase inhibitors. Several HMG-CoA reductase inhibitors or statins have been invented and approved. The chemical structures of these molecules vary widely, as shown in Figure 3, but they all contain an acyclic or a cyclized hydroxylated heptanoic acid as a side chain to the core molecule [30].

In general, statin drugs are highly effective in reducing LDL-cholesterol levels and the risk of cardiovascular disease. They are considered safe but may cause several side effects to selected individuals. This accounted for nearly 10% of all users. The main side effects of statins include muscle pain or weakness (known as *Rhabdomyolysis*), an increase in blood sugar level, an enhanced risk of type2 diabetes, loss of cognitive function, liver dysfunction, nerve pain, pancreatic and hepatic problems, as well as sexual dysfunction. Changing the type of statin medication and the dose may help to eliminate or reduce some of the side effects and provide increased tolerance [31,32,33,34,35].

### 2.2. PCSK9

Owing to the various side effects of statins and the inability of nearly 10% of users to tolerate statin drugs, there is a need for additional therapies for reducing LDL-cholesterol as alternative to statin drugs. This is particularly true for individuals suffering from hypercholesterolemia who cannot take statin drugs owing to more serious side effects [36,37]. This has led to the efforts to identify a target molecule alternative to HMG-CoA reductase for lowering LDL-cholesterol levels. PCSK9 was found to be that alternative target molecule. It is implicated in cholesterol metabolism. Since its discovery in 2003 and extensive research work in subsequent years, PCSK9 protein (also considered an enzyme) has become a prime target for the development of drugs aimed at reducing LDL-cholesterol level in the blood serum [38]. It is now well accepted that human PCSK9 (hPCSK9) [a secreted soluble protein containing 692 amino acids (aa)] is initially synthesized in the liver and then secreted into the bloodstream. It binds with LDL-R (LDL-receptor) and transports it into the lysosomal compartment where the receptor is degraded. In the absence of PCSK9, LDL-R is carried to the endosomal compartment and is ultimately recycled to the cell surface [39,40]. Therefore, the higher functional activity of PCSK9 means increased degradation of LDL-R. This ultimately results in greater accumulation of LDL-cholesterol in the blood as it will be less cleared by the receptor from the circulation in the bloodstream [41]. PCSK9 represents the third gene involved in familial hypercholesterolemia, after LDL-R and ApoB genes [42]. PCSK9 has now become the second most vital protein which has been targeted to develop cholesterol-reducing agents. It is a rational expectation that agents which block or inhibit the functional activity of PCSK9 should lower LDL-cholesterol levels in the bloodstream [43].

### 2.3. ATP-Citrate Lyase

The ATP (adenosine triphosphate)-citrate lyase (abbreviated as ACLY) enzyme is involved in cholesterol biosynthesis two steps prior to the synthesis of the intermediate molecule HMG CoA. In fact, it is a crucial hepatic cytoplasmic enzyme present in the liver which is required to produce Acetyl-Coenzyme A (Ac-CoA) from citrate. Ac-CoA is the main building block for cholesterol synthesis (see Figure 1) [44]. This tetrameric enzyme catalyzes the conversion of the citrate molecule and coenzyme A (CoA) to Ac-CoA and oxaloacetate, with the concomitant hydrolysis of ATP to ADP and phosphate. The inhibition of this enzyme is expected to block cholesterol synthesis in the very early stages, and therefore it is an important target for the development of therapeutic agents that can block cholesterol synthesis in the liver. In fact, such inhibitory molecules have been invented and shown to lower LDL-cholesterol levels by inhibiting the production of Ac-CoA [45,46].

### 2.4. Squalene Synthase (SQS)

Squalene synthase (SQS) is another crucial enzyme which acts in one of the initial steps of cholesterol biosynthesis pathway. It has been used as a potential target for the development of cholesterol-lowering drugs. This enzyme converts farnesyl pyrophosphate to squalene in the pathway. Blocking this enzyme is expected to reduce the synthesis of cholesterol. Thus, efforts have been made to design inhibitors against this enzyme. As this enzyme is implicated at a later stage in the cholesterol biosynthesis pathway, it is expected that its inhibitors may cause fewer side effects compared to those observed with statins [47].

### 2.5. Squalene Epoxidase (SQLE)

This represents another key enzyme besides SQS associated with the post-mevalonate pathway for cholesterol biosynthesis. It catalyzes the conversion of squalene to 2.3-epoxy squalene in a stereospecific manner. This is an important step in the cholesterol biosynthesis pathway and is a target for the development of cholesterol-lowering agents [48].

### 2.6. Oxidosqualene Cyclase (OSC)

Oxidosqualene synthase (OSC), also known as lanosterol synthase (LSS), is involved in the cyclization of 2,3-epoxy squalene (also called 2,3- oxidosqualene) to form the steroid or triterpene skeleton during cholesterol biosynthesis, leading to the formation of lanosterol, the crucial intermediate of cholesterol biosynthesis. This enzyme is also targeted in an effort to generate cholesterol-lowering drugs, but the efforts were not very successful [49].

### 2.7. Emopamil Binding Protein (EBP) or 3β-Hydroxysteroid Δ8-Δ7 Isomerase

Emopamil binding protein (EBP) is an important enzyme that participates in the final steps of cholesterol biosynthesis in mammals. It is also called 3β-hydroxysteroid Δ8-Δ7 isomerase. This enzyme plays role in moving the double bond from position C_8_–C_9_ to position C_7_–C_8_ in the B ring of sterol backbone in the post-squalene pathway of cholesterol biosynthesis. This enzyme has also been considered as a target to develop cholesterol-lowering agents [50]. So far, there are no reports of any drugs developed for lowering cholesterol that target any of these enzymes, namely, SQS, SQLE, OSC, and EBP. However, a few agents are considered as promising candidates.

### 2.8. Bile Acids

Another family of important biomolecules which play a key role in cholesterol regulation in the liver are bile acids, of which cholic acid and its derivatives constitute the major molecules [51]. Among many functions, one key role of bile acids is to eliminate excess cholesterol from the body. This helps to manage cholesterol balance and homeostasis. Bile acids transport cholesterol from the liver to the intestine, from where it is ultimately expelled as feces [52]. Bile acids constitute a crucial part of the entero-hepatic circulation. In addition to the role in eliminating cholesterol, excessive production and dysregulation of bile acids can cause cholesterol build-up especially in adult hepatic cells, where LDL-R is degraded by bile acids present in the entero-hepatic circulation. Interestingly, bile acids themselves are generated from cholesterol in the liver, reabsorbed by the tissues of the small intestine, and returned to the liver. This cyclic pathway is termed the entero-hepatic circulation [53,54].

### 2.9. Cholesterol Absorption

Cholesterol absorption from external sources such as food is an important event which can alter cholesterol level in the body. Thus, the cholesterol absorption process has been seen as another key target for controlling cholesterol level in the body. It is noted that cholesterol from food is absorbed and carried to the liver where the cholesterol uptake by the interior cell walls (called enterocytes) of the small intestine is highly significant. In this event, the processing of food before absorption is also crucial to ensure maximum efficiency [55,56,57]. So far, the best-known cholesterol absorption drug is Ezetimibe, which is discussed in Section 3.1.2.

### 2.10. Lipoproteins

Lipoproteins are also considered important therapeutic targets for lowering LDL-cholesterol. In fact, the mRNA of lipoproteins such as Apolipoprotein B (ApoB) has been used as a target where antisense oligonucleotide molecules have been developed that can bind and inhibit ApoB synthesis. Thus, it was demonstrated that upon binding with ApoB mRNA, such oligonucleotides produced a substrate for the enzyme ribonuclease-H, which then degraded the RNA strand. This resulted in a reduction of ApoB synthesis, leading to less production of LDL and VLDL particles and the associated cholesterol [58]. This approach of blocking protein synthesis has been successfully employed to develop lipid- and cholesterol-lowering drugs. These molecules not only inhibit ApoB synthesis but also inhibit microsomal triglyceride transfer protein (MTTP) [59]. MTTP plays an important role in the assembly and transport of lipoprotein particles and was a target for the management of lipid disorders characterized by an upregulation of ApoB-containing lipoproteins. This includes the familial chylomicronaemia and hypercholesterolemia syndromes [60].

## 3. Drugs Approved for Lowering Cholesterol

Based on the above-described targets, several cholesterol-lowering drugs have been successfully developed and approved for global use. These drugs are administered either via oral or injection routes. Irrespective of the mode of administration, all the approved drugs were found to be quite effective in lowering LDL-cholesterol levels in the blood. In addition, they were found to be safe, with a minimal or acceptable level of side effects, both in the short and long term [61,62,63]. So far, thirteen cholesterol-lowering drugs have been approved by the FDA, EMA, MHRA, and other global bodies for human use. Among them, eight are statin drugs which, as indicated before, act by inhibiting the HMG CoA reductase enzyme in the liver. The other five are non-statin drugs, among which three are PCSK9 inhibitors, one is an inhibitor of the ATP-citrate lyase enzyme, and one is a strong bile acid-binding molecule [64].

The eight approved statin drugs are (i) Lovastatin IR (Immediate Release) (also called Mevacor); (ii) Atorvastatin (also called Lipitor or Caduet); (iii) Pravastatin (also called Pravachol); (iv) Rosuvastatin (also called Crestor or Ezallor); (v) Fluvastatin (also known as Lescol or Lescol X); (vi) Pitavastatin (also known as Livalo, Zypitamag, or Sprinkle); (vii) Simvastatin (also known as Zocor, FloLipid, or Vytorin); and (viii) Lovastatin extended release variant (also known by the brand name Altoprev) (Table 1).

These statins can be divided into two broad categories, namely, Type-1 and Type-2. Examples of Type-1 statins, which are derived from natural sources, are Pravastatin, Simvastatin, and Lovastatin, whereas Type-2 statins, which are obtained by synthetic means, include Atorvastatin, Fluvastatin, Pitavastatin, and Rosuvastatin [65,66,67]. Among all statins, Fluvastatin and Pravastatin are the safest since they exhibit the fewest or no side effects compared to the other statins [68,69].

The statin market was found to be valued at USD ~15 billion in 2021 [70]. This figure is projected to become USD 18.93 billion by 2030 [Statin Market: Global Industry Analysis and Forecast (2024–2030): [https://www.maximizemarketresearch.com/market-report/global-statin-market/77560/, accessed on 11 March 2025]. In 2003, Atorvastatin, manufactured by Pfizer, became the best-selling drug in pharmaceutical history. It is interesting to note that all statin drugs are administered via the oral route and are highly effective in lowering LDL-cholesterol levels in a significant manner, depending on the dose and the nature of the drug. Among the non-statin cholesterol-lowering drugs, all three PCSK9 inhibitors are delivered via injection, while the other two are administered via the oral route.

### 3.1. Oral Drugs

#### 3.1.1. Statins

As mentioned earlier, all eight known statin drugs are orally administered, meaning that they are delivered by mouth. Statins were first discovered as LDL-cholesterol-lowering drugs by the Japanese scientist Akira Endo [71]. Though highly effective for most patients, statins are not suitable for all. Nearly 10–12% individuals cannot tolerate these drugs due to their many serious side effects or simply because they are not effective. As stated earlier, various types of statin drugs with wide-ranging chemical structures (see Figure 3) have been reported. The first statin drug, Lovastatin, was approved in 1987 and is manufactured by Merck. Later, additional statin drugs were invented by other pharmaceutical companies. In terms of structure, statins are characterized by the presence of three structural components, namely, an HMG-CoA analogue unit, a ring structure, and a hydroxylated hepta-carbon linear or cyclic chain. Most statins (except Pravastatin and, to some extent, Rosuvastatin, which are hydrophilic) are lipophilic in character. Upon taken orally, they can pass deeper into the membranes where they interact with the surrounding acyl chains. On the other hand, hydrophilic statins remain associated with the polar surface of the membrane and need the help of protein transporters to enter the cell to inhibit the HMG-CoA reductase enzyme [72]. Currently, the statin drug market is dominated by Pfizer Inc., Merck KGaA, Kowa Pharmaceuticals America Inc., Dr. Reddy’s Laboratories Ltd., Sandoz International GmbH, Novadoz Pharmaceuticals, Accord, Althera Pharmaceuticals, and Sun Pharmaceutical Industries [73,74,75].

#### 3.1.2. Non-statins

(i) Bempedoic acid (Nexletol)

**Figure 4:**
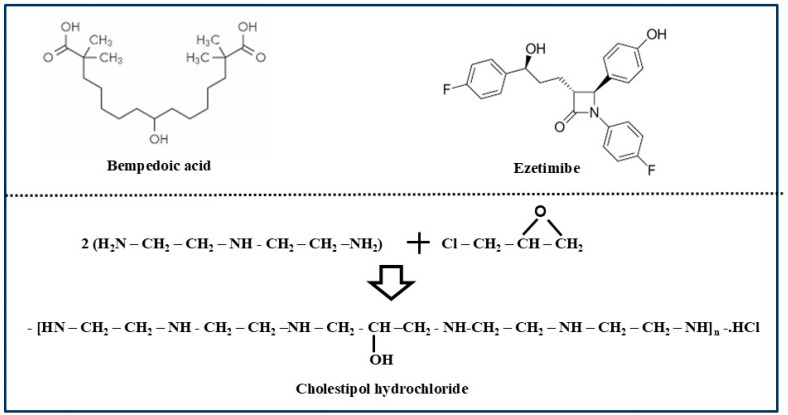
Chemical structures of three approved cholesterol lowering oral drugs Bempedoic acid, Ezetimibe and Cholestipol hydrochloride.

The best example of this class of cholesterol-lowering drug is Bempedoic acid. It is a symmetrical 15-carbon dibasic fatty acid with a central hydroxyl group (Figure 4). It is a potent inhibitor of the ATP-citrate lyase enzyme which participates in cholesterol biosynthesis in the liver [76]. This drug was developed by Esperion Therapeutics, Ann Arbour, Michigan, USA, and approved by the FDA on 21 February 2020. Extensive studies revealed that it can reduce LDL-cholesterol levels from 15 to 25%, depending on the dose. It is much less effective compared to the 40–50% reduction typically noted with most statin drugs. It acts in a manner similar to statins except that it targets a different enzyme linked to the cholesterol biosynthesis pathway [77,78].

(ii) Ezetimibe

Another drug approved under this category and administered orally is Ezetimibe (Zeha) (Figure 4), which is a strong inhibitor of cholesterol absorption in intestine tissues. It causes a reduction in the overall delivery of cholesterol to the liver, leading to the promotion of the synthesis of LDL-receptors and cholesterol uptake. It was invented by Merck and Schering-Plough, Kenilworth, New Jersey, USA, and approved on 25 October 2002, for administration via the mouth as a pill [79]. It is more effective when administered along with statins and dietary changes. It was originally discovered by Harry Davies, Gaithersburg, USA, who received the first Endo award, named after the scientist Akira Endo who discovered statins. This drug is not very efficient in lowering LDL-cholesterol, especially when used alone. It has some side effects that include stuffy nose, viral infection in the nose, and diarrhea [80,81].

(iii) Bile acid sequestrants

**Figure 5:**
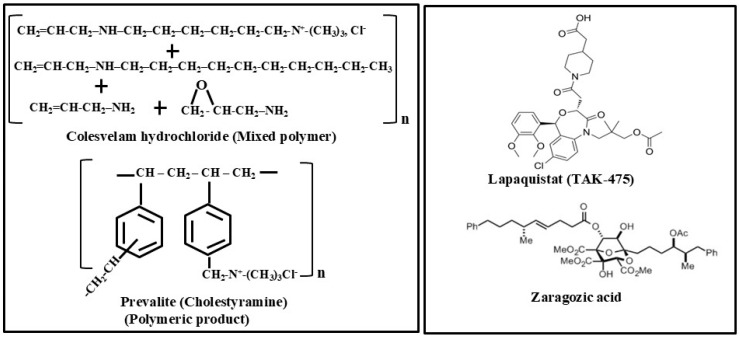
**Left** panel: Chemical structures of Bile acid sequestrants Colesvelam (also called Welchol) and Prevalite (Cholestyramine) used for lowering LDL-cholesterol. **Right** panel: Structures of Lapaquistat (TAK-475) and Zaragozic acid which are both SQS inhibitors.

These represent another important family of non-statin drugs that are administered orally as powders, tablets, or suspensions. Sequestrants are described as chemical compounds that bind to another molecule and remove it from the system. Their name comes from a Latin word meaning “*withdraw from use*”. Bile acids are produced in the liver cells by the oxidation of cholesterol. They then undergo further transformations in the intestine. Eliminating bile acids from the intestine by using bile acid sequestrants like Colestid promotes bile acid synthesis and a corresponding increase in the transformation of more cholesterol into bile acids. This leads to the reduction in cholesterol levels in plasma. In general, bile acid sequestrants are a group of ion-exchange resins that bind to certain elements of bile acids in the GI track. These drugs contain highly positively charged functional moieties that bind to the negatively charged bile acids in the intestine, thereby inhibiting their lipid dissolving activity. This helps to prevent cholesterol absorption and disrupt the entero-hepatic circulation of bile acids. Drugs that belong to this category (Table 1) are Colestid (Colestipol) (Figure 4), WelChol (Colesevelam), and Prevalite (Cholestyramine) (Figure 5) [82]. These drugs help to remove bile acids and reduce LDL-cholesterol. Colestid, originally approved in the USA in 1977, was rebranded as Colestipol hydrochloride by ANI Pharmaceuticals, Baudette, MN, USA, and then approved again on 4 April 2023 [83,84]. It can be administered orally as tablet, powder or granule form mixed with food. Colestipol or Colestid is the hydrochloride of the copolymerized product of epichlorohydrin and diethylentriamine containing varying numbers of quaternary nitrogen atoms. Colesevelam or WelChol was approved by the FDA for lowering blood cholesterol as well as glucose in 2000. It works better when combined with diet and exercise. It is manufactured by crosslinking polyallylamine with epichlorohydrin and then modifying it with bromodecane and (6-bromohexyl) trimethylammonium bromide. The bromide ions are finally exchanged with chloride ions in the final product [85,86,87]. Prevalite, also called Cholestyramine, Questran, or LoCholest, is another bile acid sequestrant drug which was first approved in 1997 for lowering LDL-cholesterol [88,89]. It was originally manufactured in suspension form by Baker Norton Pharmaceuticals Inc, Silver Spring, MD, USA.

(iv) Lapaquistat acetate/(TAK-475) and Zaragozic acid

These compounds are potent inhibitors of the squalene synthase (SQS) enzyme. Most SQS inhibitors are derived from farnesyl pyrophosphate or pre-squalene pyrophosphate structures. TAK-475, also known as Lapaquistat has been shown to be effective in lowering LDL-C. Unfortunately, they exhibit serious adverse effects and there are safety concerns. Therefore, they were never approved or brought to market [90]. Another SQS-inhibitory compound called Zaragozic acid (a natural product obtained from fungi) is considered as a promising candidate for the treatment of hypercholesterolemia. It is under study and has not yet been approved [91].

(v) Terbinafine (NB-598), Butenafine, and Naftifine

These drugs are potent inhibitors of the SQLE enzyme linked to the cholesterol biosynthesis pathway. They have been approved for the treatment of fungal infections of the nails, skin, feet, body, etc. However, they can also be used for lowering cholesterol in selected hypercholesterolemic individuals. The applications of these drugs as cholesterol-lowering agents are quite limited because of their lack of potency, safety, and other issues [92,93].

### 3.2. Nonoral (Injectable) Drugs

So far, three cholesterol-lowering drugs targeting PCSK9 have been developed and approved for global use. These drugs are required to be administered via the non-oral route, namely, through an injection under the skin (subcutaneous). They act by inhibiting the functional activity of PCSK9 or its production in the liver. These are Alirocumab (Praluent), Evolocumab (Repatha), and Inclisiran (Leqvio). The first two are both monoclonal antibodies and potent inhibitors of PCSK9 whereas the third one is a small interfering (si) oligonucleotide based on PCSK9 mRNA, which interferes with its synthesis in the hepatocytes [94,95,96].

#### 3.2.1. Alirocumab (Praluent)

This is the first PCSK9 drug approved for lowering LDL-cholesterol. It is a humanized monoclonal antibody (mAb) raised against the PCSK9 protein and developed jointly by Sanofi (Paris, France) and Regeneron Pharmaceuticals (Tarrytown, NY, USA). The drug was first approved by the FDA on 24 July 2015 and is recommended for administration only via subcutaneous injection (under the skin) every 2–4 weeks, depending on the dose. Thus, for the lower dose of 75 mg, the injection is given once every 2 weeks, whereas for the 150 mg dose, it is one injection every 4 weeks. This was an expensive treatment when it first began. The average cost of treatment now is about USD 5850 per year [97,98].

#### 3.2.2. Evolocumab (Repatha)

This is the second approved PCSK9 drug which is also a humanized monoclonal antibody against the hPCSK9 protein. It was developed by Amgen (Thousand Oaks, CA, USA) and approved on 27 August 2015 by the FDA. It also requires to be administered via an injection under the skin (subcutaneous) once every 2–4 weeks, depending on the dose. Thus, using a 140 mg dose, the drug is injected once every 2 weeks, or for the 240 mg dose, the injection is given once a month. This treatment is more expensive than the previous one. Here, the average cost per year is USD ~7000–9000, depending on the dose [99,100]. A comparative analysis between the two monoclonal-based PCSK9 drugs in terms of their efficiency, ease of administration, potency, applications, etc., has been presented in several review articles [101,102]. Another mAb of PCSK9 called “Bococizumab” from Pfizer (New York City, NY, USA) did not progress further and was discontinued in November 2016 after six studies due to poor data and weak results, according to the company’s website (https://www.sciencedirect.com/topics/neuroscience/bococizumab, accessed on 7 February 2025) [103,104].

#### 3.2.3. Inclisiran (Leqvio)

Unlike the first two PCSK9 drugs Alirocumab and Evolocumab, which are both monoclono antibody macromolecules, Inclisiran or Leqvio is not an antibody. In fact, it is a small interfering (si) double-stranded (ds) RNA molecule which was developed by Alnylam Pharmaceuticals (Boston, MA, USA), later acquired by Novartis Pharmaceuticals Inc (Basel, Switzerland) [105]. This drug was approved on 22 December 2021 by the FDA as an “add-on” treatment, especially for familial hypercholesterolemic individuals [106]. It is also administered via injection, usually twice a year. It inhibits the intracellular synthesis of PCSK9. This dsRNA, which is about 20–30 base pairs long, is composed of two strands, namely, the guide strand and the passenger strand. The guide strand is designed to act as an antisense strand which is crucial in the RNA-inhibition (RNAi) process. It consists of the target mRNA sequence which is intended for silencing. After siRNA is created, the guide strand becomes part of the RNA-induced silencing complex (RISC). It then helps to degrade PCSK9 mRNA [107]. The estimated cost per injection of this drug is about USD 6500 [108]. Two additional siRNA molecules (called SPC 5001 and BMS 844421), developed by Bristol Mayers, Lawrence-Princeton, New Jersey, USA, were also investigated but later withdrawn from the market due to various reasons.

#### 3.2.4. Mipomersen

Mipomersen (brand name: Kynamro) is a cholesterol-lowering drug which acts by inhibiting ApoB 100 (the largest and primary form of ApoB found in hepatic cells) synthesis. It is an antisense oligonucleotide derivative which blocks the translation of the gene ApoB 100. This drug was developed by Genzyme Corporation, Cambridge, MA, USA, and was approved by the FDA in January 2013 for the treatment of homozygous familial hypercholesterolemia [109,110]. It is delivered via subcutaneous injection into the abdomen, thigh, or upper arm. The drug exhibited serious side effects such as hepatotoxicity, increasing the levels of liver enzymes and liver fat. Owing to this reason, its use was heavily restricted only to treat special cases or discontinued altogether [111].

#### 3.2.5. Lomitapide

Lomitapide (brand names: Juxtapid and Lojuxta) is the other antisense oligonucleotide drug approved for lowering cholesterol in homozygous familial hypercholesterolemic individuals [110]. It is an inhibitor of ApoB 100 as well as the microsomal triglyceride transfer protein (MTTP). In fact, by inhibiting MTTP, it blocks the production of the VLDL particle and LDL-cholesterol. The drug was approved in December 2012 by the FDA but like Mipomersen, it also exhibits serious side effects including toxicity to the liver, and therefore its use is also strictly regulated [112]. An excellent review describing mRNA blocking technologies for therapeutic purpose has been published by Zhu et al. [113].

#### 3.2.6. LIB003 (Lerodalcibep)

Lerodalcibep, also known as LIB003, is a recombinant protein generated by the fusion of human serum albumin and the adnectin binding domain of human PCSK9. This injectable drug is being developed by Lib Therapeutics, Cincinnati, Ohio, USA. Following successful clinical and other studies, a biologic licence application was submitted to the FDA on 16 December 2024 for the use of Lerodalcibep for lowering of LDL-cholesterol in adults, with a monthly injection dose of 300 mg in 1.2 mL. As of December 2024, Lerodalcibep has not yet been FDA-authorized, but it is under serious consideration for approval [114,115]. If approved, it will be the fourth PCSK9-based cholesterol-lowering drugs and is expected to be less costly.

## 4. Oral PCSK9 Drugs in Progress

As noted earlier, all three PCSK9 cholesterol-lowering drugs, namely Evolocumab (Repatha), Alirocumab (Praluent), and Inclisiran (Leqvio), must be administered via injection. The fourth potential drug Lerodalcibep (not yet approved) is also designed to be injected. This is an uncomfortable route and not tolerable for many individuals due to various reasons including medical conditions. In addition, these PCSK9 drugs are all quite expensive compared to other available treatments, especially the orally administered statins. They may also cause side effects such as kidney and liver diseases. Due to these reasons, despite being more effective than statins, none of the PCSK9 drugs were able to attain the level of commercial success of statins [116,117,118]. Therefore, much attention has been drawn to the development of oral-based PCSK9 drugs for lowering LDL-cholesterol. Some progress has been made in this area although no effective product has emerged yet as a drug. Several molecules are currently under investigation as potential oral PCSK9 drugs. Despite a great deal of effort, the advancement in this field is slow but still promising. The intense interest and efforts in search of small-molecule orally active inhibitors of PCSK9 reflect a vital shift in addressing the condition of hypercholesterolemia [119]. In the sections below, we summarize the progress of these compounds in terms of their acceptance as cholesterol-lowering drugs [120].

### 4.1. AZD0780 (Laroprovstat)

**Figure 6:**
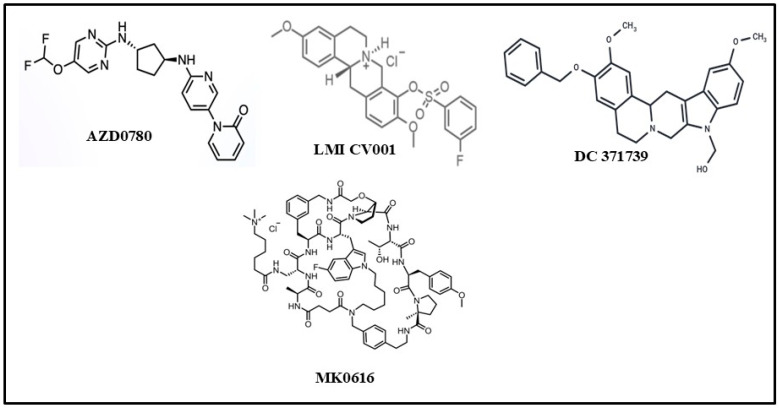
Chemical structures of several cholesterol-lowering oral PCSK9 drugs now in advanced stage of progress.

This small nonpeptide heterocyclic molecule (Figure 6) has been under investigation for its strong PCSK9 inhibitory activity. It was one of the first compounds considered a potential oral PCSK9 drug. It was manufactured by Dogma Therapeutics Inc., Cambridge, MA, USA, which was acquired by AstraZeneca, Cambridge, UK. The compound was meant for individuals with dyslipidemia that cannot be managed by statins alone. It has also been extensively studied for the treatment of hypercholesterolemia. The results of a randomized, single-blind, placebo-controlled Phase-I trial of this compound have been reported in a recent study. A reduction of 50% in LDL-cholesterol was noted when AZD0780 was given on top of rosuvastatin treatment. Moreover, a 78% total reduction in LDL-cholesterol from baseline was noted. This Phase-I trial included healthy participants receiving AZD0780 at 30 mg or 60 mg daily versus placebo (*n* = 15 for each arm) for four weeks. An additional 35 participants with hypercholesterolaemia (LDL-C > 100 mg/dL to 190 mg/dL) received rosuvastatin at 20 mg for three weeks, followed by AZD0780 at 30 mg or placebo for four weeks [121]. AZD0780 showed a significant reduction in LDL-cholesterol on top of the statin in this Phase-I trial, which is an encouraging first step. The basic structure of this molecule contains an unusual cyclopentyl-diamine group, flanked by a pyridine and a pyrazine ring system.

Following the positive Phase-I results, the process for a Phase-II studies of AZD0780 started in December 2024 and is still ongoing. This study is designed to assess the effect of AZD0780 on ambulatory blood pressure in individuals with atherosclerotic cardiovascular disease or risk equivalents and elevated LDL-cholesterol [122,123].

### 4.2. AZD8233

This is an antisense oligonucleotide molecule which is currently under investigation for its inhibition of PCSK9 mRNA translation and protein synthesis in the liver. The sodium salt of AZD8233, developed by AstraZeneca and Ionis companies, has been shown to promote the expression level of the LDL-receptor and thereby reduce the PCSK9 amount. After a successful Phase-II study (73% reduction in LDL-C), it was discontinued by the partner company Ionis, since it fell short of some thresholds for efficiencies set by the AstraZeneca company [124,125,126].

### 4.3. NNC0385-0434

NNC0385-0434 is a peptide (with undisclosed amino acid sequence) that is derived from the epidermal growth-factor-like domain A (EGF-A) of the human LDL receptor. It is a small-molecule PCSK9 inhibitor that is proposed to be taken orally. The product is currently developed by Novo Nordisk as a possible oral PCSK9 drug. A Phase-II study of this product, involving volunteers within the age group of 18- to 64-year-olds and another group with elderly population aged over 65 years, was conducted in 2021 but the data was officially reported as a synopsis in 2024 [127,128]. This Phase-II study assessed NNC0385-0434 in individuals receiving oral lipid-lowering therapy. This study demonstrated excellent LDL-cholesterol lowering efficiency during the 12-week period as well as good patient tolerance. However, the product was discontinued from further development due to portfolio considerations. Although the structure of NNC0385-0434 was not disclosed by the company, it used SNAC permeation enhancer technology. This is a method of using sodium N-(8-[2-hydroxybenzoyl] amino) caprylate (SNAC) to enhance the absorption of peptides and proteins in the stomach [129].

### 4.4. CVI-LM001

This is a small-molecule modulator of PCSK9 (see structure in Figure 6) that can inhibit the transcription of the PCSK9 gene and degrade LDL-R mRNA. It is a fluorobenzene-sulfonate derivative of Corydalin. It was manufactured by CardioVascular Innovation (CVI) Pharmaceuticals, Palo Alto, CA, USA. Initial clinical data suggest that this compound can reduce the expression of the PCSK9 gene by up to 90%. Details of the clinical data have not been disclosed. However, it exhibited good pharmacokinetics properties [130]. Further clinical studies are currently in progress.

### 4.5. DC371739

DC371739 is an indole-containing tetrahydro-isoquinoline derivative (Figure 6) which is a potent inhibitor of PCSK9. In animal studies, this compound has been shown to significantly reduce total cholesterol as well as LDL-cholesterol levels in plasma. It also exhibited positive results in the Phase-I trial in a preliminary study. This molecule was developed from a library of compounds based on the structure of tetrahydroproto-berberines (THPBs) as scaffolds. THPBs were obtained from the Chinese herbal plant *Corydalis Ambigua* which demonstrated cholesterol lowering and other biological activities [130,131]. Following positive results in the Phase-I study, it is now under clinical development for the treatment of hypercholesterolemia in a Phase-Ib/IIa clinical trial.

### 4.6. MK-0616 (Enlicitide Chloride)

MK-0616 is the most promising small-molecule PCSK9 inhibitor which is in the advanced stages of approval as an oral PCSK9 drug for lowering cholesterol. Structurally speaking, this compound is a macrocyclic peptide molecule with a molecular weight of 1612 Da (see chemical structure in Figure 6). It was discovered and developed by Merck in collaboration with Ra Pharmaceuticals, a Cambridge, USA, biotech company. It was found to be a highly potent PCSK9 inhibitor with an inhibition constant Ki of 0.00239nM [132]. It is currently in a Phase-III clinical trial that began in September 2023, following the successful completion of a Phase-IIb study [133]. It promises to be a blockbuster cholesterol-lowering drug as it exhibits a high degree of efficiency in clinical trials and of potential for applications in individuals where statins and dietary adjustments are not sufficient. It is also considered patient-friendly and safe. It can be made in an easily administered oral formulation. It has been shown to lower LDL-cholesterol in a significant manner, which is more than all other currently available tablets in the market, including bile acid sequestrants, statins, Ezetimibe, and Bempedoic acid [134,135,136].

The first study involved 90 people (10 mg–300 mg dose) vs. placebo and showed a reduction in PCSK9 level by >90% from baseline regardless of the dose of MK-0616, following 14 days of treatment [135]. In a Phase-II study, the drug demonstrated up to 60.9% reduction in LDL-cholesterol from baseline, following 8 weeks of treatment and another 8 weeks of follow-up. Moreover, in Phase-IIa and -IIb studies, this potential drug was assessed for dose requirements (how much drug should be given) and efficiency (how well the drug performs). It binds to PCSK9 with a high degree of affinity like mAbs. It was shown to be as effective as Alirocumab (Praluent), Evolocumab (Repatha), and Inclisiran (Leqvio). It is a tricyclic macrolide peptide which has been successfully synthesized via many steps using innovative chemistry in combination with mRNA display technology [137,138]. It has also been demonstrated that the gastrointestinal absorption of MK-0616 can be enhanced using a cell permeation enhancer such as sodium caprate. MK-0616 was developed in 2013 through a partnership with Ra-Pharmaceuticals, Cambridge, MA, USA, which was acquired by UCB (Union Chimique Belge) Biopharmaceuticals, Brussels, Belgium, in April 2020. This material became the molecule of the year in 2023 due to its high potential in reducing LDL-cholesterol level as an oral PCSK9 medication [https://drughunter.com/articles/mk-0616-the-2023-molecule-of-the-year, accessed on 7 February 2025]. If everything goes as expected, it will be in the global market in 2026.

### 4.7. PCSK9-Derived Peptides

It has been well established by various studies that the catalytic domain human PCSK9 (hPCSK9) comprising aa153-421 binds with the EGF-A domain of hLDL-R. This directs LDL-R to the lysosomal pathway, leading to its degradation. Normally, the receptor is transported to the endosomal compartment for recycling to the cell surface. This finding encouraged us to investigate the catalytic domain of hPCSK9 in more detail to identify the peptide segment with the highest binding affinity towards the EGF-A domain (aa314-355) of hLDL-R (an 860 aa long membrane-bound protein). Based on in vitro and cell culture as well as modelling studies, we established that three regions of hPCSK9 with disulphide bridges called Loop-1, Loop-2, and Loop-3 are of prime interest in terms of binding interaction with the EGF-A domain of hLDL-R [139,140]. Our rationale is that a well-designed peptide with a strong affinity towards the EGF-A domain of hLDL-R from the above loop segments of hPCSK9 may act as a competing peptide and thereby disrupt the hPCSK9: hLDL-R binding. Data revealed that the Loop-3-derived CP-3 peptide and its D/Y mutant variant exhibited the most potent binding affinity towards the EGF-A peptide derived from hLDL-R. They also displayed LDL-R-promoting activity, resulting in a modest uptake of LDL-cholesterol. This was demonstrated in cell culture systems using human HepG2 and Huh7 cells [139,140]. These studies were subsequently cited in several review articles [141,142]. The above-mentioned CP-3 peptide has already been patented [143,144] and is under study. This patent was acquired by the pharmaceutical company Aqur Biosciences, Inc., Westlake Village, CA, USA (https://aqurbiosciences.com/, accessed on 7 February 2025), which is now conducting clinical and other investigations for its cholesterol lowering activity. Several other closely related peptides are also included in the study [145].

## 5. Plant Sterols as Cholesterol-Reducing Agents

It was reported earlier that several sterols and related compounds isolated from plants exhibit modest cholesterol lowering activity. In fact, two plant-derived sterols, namely, Sitosterol and Sitostanol, have been used to treat the severe condition of childhood hypercholesterolemia. A clinical study with nine children suggested that Sitostanol is more effective in reducing LDL-cholesterol (33% after 3 months and 29% after 7 months of treatment) in blood serum compared to Sitosterol (20% reduction) under similar conditions. These results, based on a small cohort of children, seem to suggest that the nonabsorbable sterol Sitostanol could be a candidate drug of choice for the treatment of childhood familial hypercholesterolemia [146]. The mechanism for this bioactivity of plant sterols is not properly understood.

## 6. Future Directions

It was realized a long time ago that there is a need for non-statin-based cholesterol-lowering drugs, especially for those individuals who cannot tolerate statin drugs or exhibit serious side effects. In general, several side effects, including muscle pain, liver disease, and kidney failure, have been reported for many individuals who are on statin therapy. The alternative to statin therapy for reducing cholesterol are PCSK9 drugs, ATP-citrate lyase inhibitors, bile acid sequestrants, and cholesterol-absorbing agents. The last three types of drugs are oral based. They are either stand-alone drugs or used along with statins as they are less efficient. On the other hand, PCSK9 drugs are quite expensive, less effective than statins, and can only be administered via injection. Therefore, they are less preferable and not patient friendly [147,148]. As a result, efforts have been made to invent oral-based PCSK9 drugs. Many compounds have been investigated and are in the pipeline at various stages of studies. So far, none have been successful. However, two molecules, namely, Merck’s MK0616 and AstraZeneca’s AZD0780, are in the most advanced stages. Both have proved to be safe and effective in lowering cholesterol. These are now awaiting final approval from the FDA and other agencies. Further research worldwide in the search of additional oral therapeutic drugs directed towards PCSK9 are in progress [149]. 

## Figures and Tables

**Table 1:** List of cholesterol-lowering drugs which have been approved or are under trial. HMG-CoAR: 3-Hydroxy 3-methyl glutaryl Coenzyme A reductase; ATP-CLi: Adenosine triphosphate-citrate lyase inhibitor; GI: Gastrointestinal; PCSK9i: Proprotein Convertase Subtilisin Kexin 9 inhibitor; SQSi: Squalene synthase inhibitor; SQLEi: Squalene epoxidase inhibitor; MTTPi: Microsomal triglyceride transfer protein inhibitor; ApoBi: Apolipoprotein B inhibitor.

Number	Drug Name	Family Type	Target	Status	Delivery Route
1	Lovastatin IR (Mevacor) (Immediate Release)	Statins	HMG-CoAR	Approved	Oral
2	Atorvastatin (Lipitor/Caduet)	Statins	HMG-CoAR	Approved	Oral
3	Pravastatin (Pravachol)	Statins	HMG-CoAR	Approved	Oral
4	Rosuvastatin (Crestor/Ezallor)	Statins	HMG-CoAR	Approved	Oral
5	Fluvastatin (Lescol/Lescol X)	Statins	HMG-CoAR	Approved	Oral
6	Pitavastatin (Livalo/Zypitamag/Sprinkle)	Statins	HMG-CoAR	Approved	Oral
7	Simvastatin (Zocor/FloLipid/Vytorin)	Statins	HMG-CoAR	Approved	Oral
8	Lovastatin ER (Extended Release) (Altoprev)	Statins	HMG-CoAR	Approved	Oral
9	Bempedoic acid (Nexletol)	ATP-CLi	ATP-Citrate Lyase	Approved	Oral
10	Ezetimibe (Zeha)	Absorption inhibitor	Cholesterol absorption	Approved	Oral
11	Colestid (Colestipol)	Bile acid sequestrants	GI-elements	Approved	Oral
12	Welcol (Colesevelam)	Bile acid sequestrants	GI-elements	Approved	Oral
13	Prevalite (Cholestyramine)	Bile acid sequestrants	GI-elements	Approved	Oral
14	Alirocumab (Praluent)	PCSK9i	PCSK9	Approved	Injection
15	Evolocumab (Repatha)	PCSK9i	PCSK9	Approved	Injection
16	Inclisiran (Leqvio).	PCSK9i	PCSK9	Approved	Injection
17	LIB003 (Lerodalcibep)	PCSK9i	PCSK9	Close to approval	Injection
18	AZD0780 (Laroprovstat)	PCSK9i	PCSK9	Under study	Oral
19	AZD8233	PCSK9i	PCSK9	Discontinued	Oral
20	MK-0616 (Enlicitide chloride)	PCSK9i	PCSK9	Close to approval	Oral
21	NNC0385-0434	PCSK9i	PCSK9	Under study	Oral
22	CVI-LM001	PCSK9i	PCSK9	Under study	Oral
23	DC371739	PCSK9i	PCSK9	Under study	Oral
24	Lapaquistat acetate (TAK-475)	SQSi	SQS	Discontinued	Oral
25	Zaragozic acid	SQSi	SQS	Under study	Oral
24	Terbinafine (NB-598)	SQLEi	SQLE	Under study	Oral
25	Mipomersen	ApoBi	ApoB	Approved	Injection
26	Lomitapide	ApoBi/MTTPi	ApoB/MTTP	Approved	Injection

## Abbreviations

aa	Amino acid
ACLY	Adenosine triphosphate-citrate lyase
ADP	Adenosine Diphosphate
CVD	Cardiovascular disease
CHD	Congenital heart disease
Da	Dalton
DALYS	Disability-Adjusted Life Years
DHCR24	Dehydrocholesterol reductase24
EBP	Emopamil binding protein
EGF-A	Epidermal Growth Factor like-domain A
EMA	European Medicines Agency
FDA	Food and Drug Administration
GI	Gastrointestinal
h	Human
HMG-CoA	3-Hydroxy-3-Methyl Glutaryl Coenzyme A
IDL	Intermediate-density lipoprotein
IR	Immediate Release
LDL	Low-density lipoprotein
mAb	Monoclonal antibody
MHRA	Medicines and Healthcare products Regulatory Agency, UK
mRNA	Messenger Ribonucleic Acid
MW	Molecular weight
OSC	Oxidosqualene cyclase
PCSK9	Proprotein Convertase Subtilisin Kexin9
RISC	RNA-induced silencing complex
SNAC	Sodium N-(8-[2-hydroxybenzoyl] amino) caprylate
SQLE	Squalene epoxidase
SQS	Squalene synthase
tBu	Tertiary butyl
THPBs	Tetrahydroproto-berberines
VLDL	Very low-density lipoprotein
